# Early Management of Suspected Cervical Spine Injury: A Real-World Insight From a London Major Trauma Centre

**DOI:** 10.7759/cureus.87217

**Published:** 2025-07-03

**Authors:** Muhammed Monjur Ahmed, Shehryaar Kiani, Mohammed Tanvir Shah, Ayman El-Zanaty, Muhammad Subed Ali

**Affiliations:** 1 Trauma and Orthopaedics, Royal London Hospital, London, GBR; 2 Trauma and Orthopaedics, Aintree University Hospital, Liverpool, GBR; 3 Trauma and Orthopaedics, Southend University Hospital, Southend-on-Sea, GBR; 4 Trauma and Orthopaedics, Royal National Orthopaedic Hospital, London, GBR; 5 Trauma and Orthopaedics, Basildon University Hospital, Basildon, GBR

**Keywords:** cervical spine fracture, emergency neurosurgery, major trauma centre, spine examination, trauma care protocols

## Abstract

Cervical spine trauma represents a critical subset of blunt injuries, accounting for 2-3% of cases and over half of spinal cord injuries. This retrospective narrative review from the Royal London Hospital analyses the initial management of such injuries, emphasising protocol-led care involving spinal protection (British Orthopaedic Association Standards for Trauma (BOAST), Advanced Trauma Life Support (ATLS), National Institute for Health and Care Excellence (NICE)), clinical assessments (Glasgow Coma Scale (GCS), American Spinal Injury Association (ASIA)), and imaging (multidetector computed tomography (MDCT), magnetic resonance imaging (MRI)). Multidisciplinary involvement, particularly anaesthetics, intensivists, and spinal surgeons, was key to optimising outcomes. MDCT showed excellent diagnostic accuracy, and early surgical decompression within 24 hours, as supported by the Surgical Treatment of Acute Spinal Cord Injury Study (STASCIS), correlated with better neurological recovery. Steroids and other pharmaceuticals have either ceased use or are currently avoided due to adverse effects. Overall, timely imaging, coordinated care, and evidence-based interventions are central to effective cervical spine trauma management.

## Introduction and background

The cervical spine is a precarious anatomical structure, accounting for 2-3% of blunt trauma cases and 55% of spinal cord injuries [1]. Victims of such injury can be left debilitated and with a huge compromise to their quality of life. For instance, it can pose a significant risk of sensory and motor neurological impairment. Hence, early, safe, and effective management is essential to prevent secondary injury [2-5], further deterioration, and long-term disability.

The Royal London Hospital, a Major Trauma Centre (MTC), admits approximately 15 spine fractures per week [2]. Standardised protocols such as the Advanced Trauma Life Support (ATLS) [3], the British Orthopaedic Association Standards for Trauma (BOAST) [4], and the National Institute for Health and Care Excellence (NICE) guidelines [5] ensure a systematic approach to cervical spine assessment, immobilisation, and early specialist intervention.

This paper explores the initial assessment and management of cervical spine injuries in the Emergency Department (ED), evaluating the cervical spine injury algorithm, including anamnesis, clinical assessment, physical examination, choice of imaging modality, and onward specialist intervention.

General initial trauma management in an MTC

With regard to a routine trauma patient, their arrival is prompt with the London Ambulance Service (LAS) or London Helicopter Emergency Medical Service (HEMS). A thorough ‘hands-off the patient’ handover is completed, which includes the date of injury, mechanism, past medical history, regular patient medication, allergies, and interventions (such as the amount of analgesia given) conducted by the pre-hospital team handing the patient over to the hospital. This handover is overheard by the trauma team and its trauma team leader (TTL). The trauma team then manages the patient using a systematic A-E approach (also known as the primary survey) [1] to ensure that any life-threatening injuries are managed as fast as possible, with the assistance of cardiorespiratory monitoring to guide initial management. Once the patient is hemodynamically stable, imaging is key to identifying further injuries or better delineating injury patterns and effects. Next, a secondary survey by the orthopaedic team is conducted to obtain the full scope of injuries, with further input from the suitable team to manage their injuries.

Spinal clearance in a trauma patient

According to the BOAST guidelines, all hospitals managing trauma patients should have a spinal protection protocol in place, and all patients with significant blunt trauma above the clavicles must be presumed to have an unstable injury to their cervical spine. The incidental risk is approximately 2% in conscious patients and up to 34% in an unconscious patient [2].

Spinal immobilisation includes a hard cervical collar, three head blocks, and spinal alignment maintenance during transfer [6]. Research supports rigid spinal precautions to maintain alignment and prevent excessive movement, and failure to adhere to spinal clearance policies can result in deterioration of the initial injury [7-8].

The four-person log roll is the method primarily used in the National Health Service (NHS). At least four people are needed for logrolling a patient to remove the spinal board and/ or examine the patient’s spine, according to ATLS guidelines [4]. One person controls the head, two people control the body, and the fourth person examines the spine. This becomes a five-person log roll if there is limb injury [4].

Clinical examination

The clinical examination for a suspected cervical spine injury includes three main objectives. Firstly, an assessment of consciousness with the use of the Glasgow Coma Scale (GCS) [9,10], to objectively describe the extent of impaired consciousness in all trauma patients. Secondly, a neurological examination (using the American Spinal Injury Association (ASIA) classification) to determine sensory and motor deficit, with consideration of symptomology in line with incomplete and complete spinal cord syndromes. Thirdly, a ‘look, feel, and move’ approach to physical examination. 

The clinical examination follows a ‘look, feel, and move’ approach: 'look' for asymmetry or deformities in the cervical spine, then 'feel' for midline cervical spine tenderness (clinicians should not attempt to reduce an obvious deformity [4]), and finally, 'move' (if safe) to assess active range of motion (assessing flexion, extension, lateral flexion, and rotation) of the cervical spine.

Once these are complete, according to the Canadian C-Spine Rule (CCR) and the National Emergency X-Radiography Utilisation Group (NEXUS) guidelines (Table 1), the cervical spine is cleared clinically [11-14], and immobilisation can be removed to avoid pressure sores and pulmonary complications [2]. Otherwise, a radiological assessment is required.

**Table 1:** Criteria for clearance of low-risk patients with cervical spine injury, according to the CCR and NEXUS [7]. NICE have adopted this into their guidelines [1] GCS: Glasgow Coma Scale; NICE: National Institute for Health and Care Excellence Table created by author Muhammed Monjur Ahmed, with the use of references stated

Canadian C-Spine Rule (CCR) [14]	National Emergency X-Radiography Utilisation Group (NEXUS) [15]
Normal conscious level (GCS 15)	Normal conscious level (GCS 15)
No evidence of intoxication	No evidence of intoxication
No painful, distracting injury	No painful distracting injury, such as blunt trauma to the head or neck, high fall greater than one metre or five stairs, high energy, and penetrating trauma [13]
No high-risk factors (e.g. >65 years old, dangerous mechanism of injury, paraesthesia in extremities)	No focal neurological deficit, for example, paraesthesia in the upper or lower limbs
Presence of low-risk factors allowing safe motion assessment (e.g. low-velocity trauma, seated position, post-traumatic ambulation, delayed onset of neck pain, no midline cervical spine tenderness)	No posterior midline cervical tenderness
Ability to actively rotate neck 45 degrees left and right	

The pitfalls of a neurological examination are pain and an altered level of consciousness. In such patients, it is important to document clearly, repeat all three parts of the exam multiple times, and ensure an adequate amount of analgesia is provided. The clinical examination findings should be paired with imaging to confirm the diagnosis of a cervical spine fracture.

Key findings, like the level of consciousness and neurological deficits (using the ASIA scoring), with a confirmed cervical spine fracture, guide decisions regarding immobilisation and monitor signs of improvement/deterioration to assist with rehabilitation, and if abnormal neurological signs consistent with spinal cord injury are found, an immediate discussion with spine surgeons must occur [3] to ensure prompt further management.

## Review

Imaging modalities: plain radiographs, computed tomography, and magnetic resonance imaging (MRI)

Plain radiographs are the simplest method of imaging; however, they are beneficial in the initial management of a suspected cervical spine injury, due to their cheap, fast, and informative properties. Plain radiographic films for visualisation and decision making of management of the cervical spine should capture the region from the occiput to T4, including anteroposterior (AP), lateral, and open-mouth odontoid views. If the lateral film does not clearly show all seven cervical vertebrae, a swimmer’s view of the lower cervical area and upper thoracic area should be obtained. The open-mouth odontoid views should include the entire odontoid process, along with the right and left C1-C2 articulations. The AP cervical spine view is particularly useful in detecting unilateral facet dislocations, especially when minimal to no displacement is evident on the lateral film [4].

According to the ATLS guidelines [4], key radiographic findings include misalignment of the posterior aspect of the vertebral bodies, which correspnds to the anterior extent of the vertebral canal, fractures or bony deformities affecting the vertebral body or its processes, widening of the interspinous space at a single level, narrowing of the vertebral canal, and enlargement of the prevertebral soft tissue space. When these films are of good quality and accurately interpreted, unstable spine injuries can be identified with a sensitivity exceeding 97% [4]. In such cases, the cervical collar may be removed to obtain flexion and extension views.

A multidetector computed tomography (MDCT) is a type of scan using multiple detectors to produce thin slices in a single rotation; hence, it is effective in determining occult bony injury. MDCT is the preferred first-line imaging modality, providing high sensitivity (98.1%) and specificity (98.8%) for cervical spine fractures [15]. MRI is reserved for cases with suspected spinal cord injury, ligamentous injury, or soft tissue compromise [3]. MRI benefits from the use of T2-weighted imaging sequences that can detect other pathologies that can cause long-term neurological compromise, such as intramedullary cord edema, hemorrhage, or myelomalacia. They also benefit from short tau inversion recovery (STIR) sequences, which are particularly adept at identifying ligamentous injuries, which may indicate spinal instability even when CT scans do not show any fractures. Therefore, MRI is crucial for patients with persistent neurological symptoms, despite normal CT results (also known as spinal cord injury without radiographic abnormality (SCIWORA)), as well as for obtunded or intubated individuals who cannot be assessed clinically [14-18]. MRI is also employed for pre-surgical planning, especially in situations involving cord compression or sequestered disc material. 

However, it is generally not utilised during the acute resuscitation phase due to constraints such as limited access, lengthy scan durations, and the requirement of a stable patient. According to the BOAST and NICE guidelines, MRI should be used selectively in cases where the clinical situation remains ambiguous after initial radiological assessment or when spinal cord injury is suspected despite normal bony imaging. Plain radiographs are less sensitive than MDCT but can assist in initial assessment when CT is unavailable.

Overall, a senior radiologist must review and report spinal clearance imaging before spinal protection precautions are discontinued, as per the BOAST guidelines [2]. The cervical collar should not be removed until a thorough neurological assessment, cervical spine evaluation, and imaging confirm the absence of injury [4]. Additionally, an initial report of cervical spine clearance imaging should be made available before the patient is discharged from the emergency department, with a definitive report within 18 hours of injury, according to the BOAST guidelines [2].

The flexibility of the spine predisposes it to injury. Those with painful, distracting injuries should be suspected of having spinal trauma, as per the CCR and NEXUS guidelines. To confirm findings, imaging is key in the initial management of cervical spine fracture patients.

Specialist involvement in the initial management

Anaesthetists

Approximately one-third of patients with upper cervical spine injuries (above C3) die at the scene from apnoea, caused by loss of central innervation of the phrenic nerves [4]. Thus, once a patient arrives at the emergency department with suspected cervical spine trauma, hypoventilation and airway are the foremost concern in the initial management and are managed with help from difficult airway guidelines [19] for patients with suspected or confirmed cervical spine injury.

Clinicians must evaluate patients' airway patency and ventilation adequacy quickly and precisely. Prompt action to enhance oxygenation and lower the risk of additional ventilatory compromise if injury is detected or suspected is paramount, with the use of end-tidal CO_2_ measurement and pulse oximetry [3].

Securing the airway in patients with suspected cervical spine injury presents a critical challenge, as any neck movement may exacerbate spinal cord damage. Established difficult airway recommendations [19] should be followed for airway care, with a focus on keeping the cervical spine immobilised at all times, through manual in-line stabilisation (MILS). Although video laryngoscopy is frequently chosen over direct laryngoscopy because it provides better glottic vision with less head extension, rapid sequence induction (RSI) is still the gold standard in emergency situations. In anticipated difficult airways, awake fibreoptic intubation is the gold standard, allowing spontaneous respiration and real-time visualisation while preserving neck alignment. Supraglottic airways (e.g. i-gel, laryngeal mask airways (LMA)) may serve as temporary solutions or rescue adjuncts. Early consideration of a surgical airway (cricothyroidotomy) is necessary when intubation fails and ventilation cannot be achieved. It is critical to adhere to difficult airway algorithms (DAS guidelines) to minimise both hypoxic injury and secondary spinal insult.

Intensivists

Cervical spinal cord injuries above T6 can lead to neurogenic shock (hypotension and bradycardia) due to loss of sympathetic tone (3). Vasopressors (phenylephrine, dopamine, or norepinephrine as recommended by NICE [5]) may be required for resuscitation, as fluid administration alone is often insufficient [3,20-23]. An extensive analysis of 490 isolated spinal cord injury cases from the Trauma Audit and Research Network (TARN) revealed that typical neurogenic shock occurred in only 19.3% of cases [22]. In contrast, a retrospective study conducted at a higher-volume level 1 trauma centre found that 31% of patients (19 out of 62) with high cervical spine injuries (cord level C1-C5) experienced neurogenic shock [24]. Also, some studies [11,24] have suggested that hypotension, resulting from neurogenic shock, may worsen the severity of spinal cord injury. Thus, initial resuscitation is key for the initial management of cervical spine fractures; hence, intensivists are involved early once the patient is brought in and form part of the trauma team.

Cervical spine fractures with associated spinal cord injury require a multidisciplinary approach, including anaesthetists and intensivists, to manage the airway and circulatory instability. Urgent discussion with orthopaedic spine surgeons is critical to prevent permanent neurological deficits.

Orthopaedic Spine Surgeons

Once a cervical spine injury is confirmed, patients are referred for surgical or non-surgical management. Instability of a cervical spine fracture is determined using the AO Spine Classification System [25], developed in 2018. The AO Upper Cervical Injury Classification helps classify fractures with knowledge of the upper cervical spine fracture overview (with assistance of imaging), patients’ neurology, and modifiers, including vascular abnormalities, patient-specific factors, and risks of non-union if left untreated.

The decision to proceed with surgery is typically based on three tenets: mechanical instability, neural compression, and failure of conservative management. Mechanical stability refers to the failure of the vertebral column's ability to maintain alignment under physiological loads, often due to ligamentous injury (diagnosed by MRI) or multicolumn fractures [26]. Neural compression, evident on CT or MRI, may result from retropulsed bone fragments, disc herniation, or hematoma and is an indication for urgent surgical decompression. Conservative/non-operative management may fail in cases of poor compliance, progression of deformity on serial imaging (non-union), or worsening neurology. 

The choice of surgical technique depends on injury pattern, neurological status, and the surgeon's expertise. The most common procedure is anterior cervical discectomy and fusion (ACDF), particularly for subaxial injuries with compromise of the anterior column. ACDF allows decompression of the spinal cord and its nerve roots and stabilisation via anterior plating and interbody grafting. Posterior cervical stabilisation is indicated for injuries involving the posterior elements (e.g. laminar fractures, ligamentous disruption, or facet dislocations) and may involve lateral mass or pedicle screw fixation. Combined anterior-posterior approaches may be required for complex or highly unstable injuries. In upper cervical injuries (C1/C2 fractures), surgical options include posterior C1-C2 fusion or occipitocervical fusion, depending on the location and pattern of instability. 

In cases of unstable cervical spine fracture, operative intervention is usually required [27], though the optimal timing of surgical treatment remains debated. The Surgical Treatment of Acute Spinal Cord Injury Study (STASCIS) found that early decompression (<24 hours) for traumatic cervical cord injury was associated with lower complication rates and better neurological outcomes at one year, compared with delayed surgery. Urgent surgical intervention is generally required in cases of progressive neurological deterioration [28].

In selected cases, especially in frail patients or those with minimally displaced fractures, non-operative management may be appropriate. This typically involves immobilisation with a rigid cervical collar (Miami J) or halo vest, particularly for stable injuries without neurology. However, note that a halo vest can be associated with complications, such as pin site infection, and poor tolerance and may delay mobilisation, so monitoring and education are key. All in all, the decision for surgical vs non-surgical management must be tailored based on patient factors, radiological findings, and functional goals of the patient. 

Pharmaceuticals in the initial management of cervical spine trauma

Methylprednisolone was previously recommended for spinal cord injury based on the National Acute Spinal Cord Injury Study (NASCIS) trials [28]. However, BOAST and NICE guidelines do not advocate steroid use, due to a lack of convincing evidence and increased pneumonia and sepsis risk [5, 29]. Tranexamic acid (TXA), a synthetic antifibrinolytic drug, is safe to use for isolated spine trauma [30] and effectively reduces blood loss by inhibiting plasmin-induced fibrin breakdown.

Many drug trials [31] with riluzole [32], erythropoietin [33], minocycline [34], atorvastatin [35], and neurotropic growth factors [36] have taken place, with no resultant change yet to justify the use of such pharmaceutical agents in the initial management of suspected or confirmed spinal cord injury guidelines (Table 2).

**Table 2:** Pharmaceutical adjuncts in cervical spine trauma NASCIS: National Acute Spinal Cord Injury Study; RISCIS: Riluzole in Spinal Cord Injury Study; RCT: randomised controlled trial; GM1 glucoside: monosialotetrahexosylganglioside Table created by author Muhammed Monjur Ahmed with the respective references listed

Agent	Mechanism of action	Trial/status	Outcome summary
Methylprednisolone [28]	Glucocorticoid, anti-inflammatory, reduces lipid peroxidation	NASCIS I–III (1984-1998)	Initial benefit reported in NASCIS III; now not recommended due to high infection/sepsis risk
Riluzole [32]	Inhibits glutamate release, reduces excitotoxicity	RISCIS trial (Phase I/II); RISCIS Phase III ongoing	Early trials showed motor recovery benefits, awaiting final results
Minocycline [34]	Broad-spectrum tetracycline; anti-apoptotic, anti-inflammatory	Canadian Phase II RCT (2012)	Some benefit in motor recovery; no large-scale trial yet
Erythropoietin [33]	Anti-apoptotic, enhances perfusion and neuro-regeneration	Phase II RCTs (Italy, 2014)	Early promise; inconsistent data; further trials needed
Atorvastatin [35]	Statin, anti-inflammatory and antioxidant	Experimental trials	Limited human data, small animal models suggest benefit
Neurotrophic growth factors (GM1 ganglioside) [36]	Neuronal membrane stabilisation, enhances regeneration	No recent large RCTs	Mixed results in older trials; no current recommendation
Neurotrophic growth factors (intrathecal stem cells) [36]	Promote neural repair and remyelination	Phase I/II trials underway	Safety demonstrated; efficacy under investigation

## Conclusions

In conclusion, effective initial management of cervical spine injuries follows a systematic approach prioritising airway stability, spinal immobilisation, appropriate imaging, and timely specialist referral. Additionally, adherence to standardised trauma protocols, such as BOAST, ATLS, and NICE guidelines, ensures best practice of the initial management in trauma hospital settings, minimises secondary injury risks, and optimises patient outcomes. As guidelines and evidence evolve, future research may refine treatment algorithms to further reduce complications and improve long-term recovery for cervical spine fractures.
